# Healthchain: A novel framework on privacy preservation of electronic health records using blockchain technology

**DOI:** 10.1371/journal.pone.0243043

**Published:** 2020-12-09

**Authors:** Shekha Chenthara, Khandakar Ahmed, Hua Wang, Frank Whittaker, Zhenxiang Chen

**Affiliations:** 1 Institute for Sustainable Industries and Liveable Cities, Victoria University, Melbourne, Victoria, Australia; 2 School of Information Science and Engineering, University of Jinan, Jinan, China; Charles Sturt University, AUSTRALIA

## Abstract

The privacy of Electronic Health Records (EHRs) is facing a major hurdle with outsourcing private health data in the cloud as there exists danger of leaking health information to unauthorized parties. In fact, EHRs are stored on centralized databases that increases the security risk footprint and requires trust in a single authority which cannot effectively protect data from internal attacks. This research focuses on ensuring the patient privacy and data security while sharing the sensitive data across same or different organisations as well as healthcare providers in a distributed environment. This research develops a privacy-preserving framework viz Healthchain based on Blockchain technology that maintains security, privacy, scalability and integrity of the e-health data. The Blockchain is built on Hyperledger fabric, a permissioned distributed ledger solutions by using Hyperledger composer and stores EHRs by utilizing InterPlanetary File System (IPFS) to build this healthchain framework. Moreover, the data stored in the IPFS is encrypted by using a unique cryptographic public key encryption algorithm to create a robust blockchain solution for electronic health data. The objective of the research is to provide a foundation for developing security solutions against cyber-attacks by exploiting the inherent features of the blockchain, and thus contribute to the robustness of healthcare information sharing environments. Through the results, the proposed model shows that the healthcare records are not traceable to unauthorized access as the model stores only the encrypted hash of the records that proves effectiveness in terms of data security, enhanced data privacy, improved data scalability, interoperability and data integrity while sharing and accessing medical records among stakeholders across the healthchain network.

## 1 Introduction

With the advancement in information and communication technology (ICT), most of the healthcare organizations paved the way for Electronic Health Records (EHRs) from paper based records. EHR, Electronic Health Data (EHD), Electronic Medical Records (EMR) are digitalized patient records encompassing a huge variety of medical data such as medical histories, demographic information, laboratory test reports and other sensitive patient personal information including social security number and credit card information [1]. The large scale generation and rampant usage of health information in the big data era increases the role of cloud networks not only to house the large amount of data but also to facilitate its access across the Internet [2–5]. Moreover the lion’s share of medical data is extremely sensitive and confidential, its storage on third party centralized servers naturally increases the privacy and security vulnerabilities that leads to several attacks includes DDoS attack [6] and Ransomware attacks that have greater ramifications beyond financial or privacy breach [7, 8]. Considering the vulnerable nature of healthcare data in the public domain and unavailing security frameworks, there is an imminent need to protect the data and devise a secure, efficient and effective mechanism to facilitate share and access of data among various stakeholders [2, 9, 10]. Blockchain technology has a large potential to bring significant efficacies to financial transactions, global supply chains, asset ledgers, healthcare and decentralized social networking.

Blockchain is one of the solutions to overcome most of the limitations in the existing distributed environment by introducing a patient centered electronic health system namely Patient Controlled Electronic Health Record System (PCEHR), in which the patient is the universal consent provider of their data to all stakeholders except in emergency situations. Blockchain is a public, decentralized, append-only, immutable digital ledger with a time stamped series of transactions called blocks that are linked to form a chain that are secured by means of Public Key Encryption cryptographic principles [11, 12]. Since the blocks are linked, the data once recorded cannot be altered retroactively without the modification of all subsequent blocks. A cryptographic one way hash function (e.g. SHA-256) is also applied to the blocks to ensure immutability, anonymity and tamper resistant structure for the blocks [13]. Moreover, blockchain uses the consensus protocol mechanism to generate, update and validate transactions for ensuring the security and also employs scripting code to run intelligent smart contracts [14, 15]. In particular, our blockchain network resolves the challenges related with interoperability, scalability, integrity, security and privacy concerns in the health care data systems and delivers a comprehensive clinical care. Our research exploits the inherent properties of blockchain to build a potential framework that fulfills the health care use-cases and supports the shift from institution-driven-interoperability to patient-centric-interoperability. This work employs Hyperledger fabric [16] as the permissioned blockchain solution that provides a framework for securing the interactions within the entities in the healthchain network.

### 1.1 Motivation

Major drawback in the current system is that since the healthcare records are stored in centralized databases in silos, healthcare data becomes an extremely tempting target for the attackers. Several research studies showed that centralization increases the security risks and requires trust in a single authority. The centralized databases can leave us vulnerable to attacks that escalates in to cyber threats ranging from the recent Ransomware attack [17] to the Equifax attack [18] which hinders the privacy and security of EHRs. Lack of Interoperability in EHR is another main issue faced by healthcare industry today. Health data in the prevalent systems is fragmented and is challenging to share with healthcare providers or stakeholders due to their varying formats and standards. This defines that it is difficult to aggregate and examine patient data that prevents the efficacy of the EHR sharing in emergency situations. Other significant concern faced by health records housed in cloud servers is from internal attacks where the people with authorized credentials within organizations access data such as database administrators or key managers are attackers, which is considerably worse than the external attacks. Further, when the EHR is deleted from the database of the hospital, the record can be permanently lost which is another issue that requires deliberate attention. It is essential to have a tamper proof system inaccessible to all except to authorized stakeholders. A traditional database system addresses these requirements only in part and thus alternative technologies need to be explored. In addition, conditional access of patient records to various physicians, laboratories and pharmacies rather than full public access is also crucial to preserve privacy of patient records. Moreover, in the existing system, patients are not in complete control of the health records since it is managed by the service providers. Because of the incessant increase of healthcare data, secure storage and scalability of medical records are a major concern. Considering the vulnerable nature of healthcare data, efficient data sharing between the stakeholders in a public domain is a complicated task. Despite the great features the existing healthcare industry provides, it fails to provide an efficient way to store, share and analyze the health data in a globally unified way. The available privacy preserving mechanisms are inadequate to ensure foolproof security for the seemly management of EHRs in the cloud [19].

In this research work, we propose a blockchain framework based on Hyperledger Fabric [16] and designed a system which can be used for efficient data sharing, health records management and systematic access control. Consequently, this research introduces a permissioned patient-centric blockchain namely Healthchain for EHRs that eliminates most of the bottlenecks and evades the likelihood of single point of failure in existing systems by introducing a distributed ledger platform. The interoperability challenges in healthcare is resolved by the healthchain framework in the way it is built. i.e. Healthchain framework stores the patient history by syncing records in different formats by accessing data via REST server API by employing self-governing and constantly executing smart contracts in the framework. And also the patient has complete control over the healthcare records by providing access and identity permissions to the authorized stakeholders by employing appropriate encryption mechanism and access control permission rules. Moreover, the immutability of health records is also achieved by cryptographically storing the data inside i.e. by storing hash values of data in the blockchain and storing encrypted healthcare records in the offchain IPFS database that makes the framework tamper resistant. Healthchain is a decentralised framework in a way that nobody can tamper the records as the data transactions are linked and a consensus of stakeholders need to agree for adding data in the network. Our system contributes to the healthcare by addressing most of the challenges with data privacy, security, interoperability, scalability, trust, immutability and data integrity.

### 1.2 Contribution

We present the structure and functionality of a permissioned blockchain based architecture called Healthchain by employing Hyperledger Fabric to securely and scalably share healthcare records to preserve patient privacy, deliver efficient permission management among stakeholders for enhancing collaborative clinical decision support and comprehensive patient care. The main contributions of this paper are summarized as follows:

- Initially, this research builds a patient centric interoperability healthchain framework in which patients will have entire control over their medical records that maintains security, privacy, scalability and integrity of the e-health data. The Healthchain framework is built on Hyperledger fabric, a permissioned distributed ledger solutions by utilizing Hyperledger composer and stores EHR in InterPlanetary File System (IPFS) to build this private healthchain network. Because of its decentralized property this framework ensures no single point of failure and also changes to the blockchain will be visible to the participants of the healthchain network that are immutable.
- To maintain the efficiency and scalability of the blockchain, this research stores only the hash of health records on chain and actual huge data is stored after encryption in the off chain storage framework in IPFS, the decentralized storage. Furthermore, the proposed healthchain framework only allows true records to be added on blockchain which is authenticated by the consensus and the access to the health records are given only based on user permission. Moreover, the data stored in the IPFS will be encrypted by using a unique public key encryption cryptographic algorithm to create robust blockchain solutions for electronic health data.
- Our research design focuses on patient-centric approach where the patient has the complete control to provide access permissions to the authorized stakeholders and does not involve any form of mining incentives beyond the efficient use of the system. This framework developed a working prototype in which the blockchain technique is analyzed and also unravels the possibility of blockchain in healthcare solutions.

The remainder of the paper is structured as follows. Section 2 discusses the related work, whereas section 3 discusses the preliminary components, section 4 explains cryptographic process and architecture of proposed framework; section 5 provides the prototype implementation of the framework; section 6 demonstrates the results; section 7 discusses the analysis and discussion of the proposed framework; and section 8 as conclusion.

## 2 Related works

This section summarizes the related works pertaining to secure storage and efficient access control schemes implemented in e-healthcare using blockchain technology. For permissionless or public blockchains such as Bitcoin [11] and Ethereum [20], anyone can join as a node in the network since public blockchain doesn’t have any network barrier. Moreover, transactions in public chains are transparent and open though anonymity is maintained but is less desirable in healthcare industry which manages sensitive health records. In contrast with the public blockchain, permissioned blockchain or private blockchain such as Hyperledger fabric adopts access control mechanism for determining the addition of a new node to the network. However, the previous studies come with the inadequacy of requisite of mining incentives in the form of ether for performing transactions in healthcare arena.

Several tamper-proof mechanisms are proposed using blockchain technology [21] as shown in Table 1. Yue et al [22] proposed the first scheme using blockchain in healthcare industry that mentioned a Healthcare Data Gateway, the possibility of data sharing on a private blockchain that facilitates patients to manage their health data without any violation of privacy or security. However, this scheme is needed to access data without explicit patient agreement and do not allow any family member to allow data access in emergency situations. Also as the e-health data is growing, scalability is a major issue due to data storage on chain which further leads to the centralization of the blockchain. MedRec [23] is the first functioning prototype in healthcare based on permissionless blockchain implementation utilizes the Ethereum smart contract functionality for the intelligent representation of the medical records which are stored in individual nodes in the network. However, mining mechanisms are required to sustain the distributed ledger; also scalability is considered as another concern with the rise of EHR every second. Other blockchain implementation by Ivan et al [24] is the creation of a blockchain based on Electronic Health Records in which healthcare data is encrypted and stored publicly. Some other techniques has proposed against malicious adversaries [25]. Another blockchain approach in healthcare is by the Medchain, a permissioned network of stakeholders to facilitate healthcare data sharing between hospitals, patients and pharmacies [26]. However, the model storing actual data on chain have significant privacy and scalability issues. A decentralised approach proposed in which the encrypted data is stored off chain and the blockchain layer enforces access control mechanisms by Zyskind et al [27]. The data privacy is a crucial issue with this blockchain technique as the patient’s metadata is exposed, which exposes all other information. All the approaches discussed here lack security, privacy and scalability concerns that needs to be addressed [28].

**Table 1:** Existing techniques using blockchain technology in healthcare.

Ref.	Addressed Challenges	Challenges to be solved
[23]	Access control, Data Integrity, Interoperability	Data scalability
[26]	Data Sharing, Data Integrity	Data privacy, Data scalability
[29]	Access control, Interoperability, secure data transfer	Data storage
[30]	Data Integrity, Access Control, Interoperability	Collective decision making
[31]	Data Integrity, Data Security	Data Storage and Scalability
[32]	Interoperability, Access Control	Data Storage and Sharing
[33]	Data Integrity, Global data access	Authentication, Interoperability
[34]	Interoperability, Provenance	Data Storage and Security
[35]	Interoperability	Scalability, Data privacy and security
[36]	Data privacy, Data security	Interoperability, Data scalability

Ancile [29] is another permissionless blockchain structure which utilizes Ethereum based smart contracts that stores hash value of the data references on blockchain for secure, interoperable and efficient access control and employs advanced cryptographic techniques such as proxy re-encryption [37] for the secure transfer of the medical records. Nevertheless, Ancile has technical difficulties such as rewriting of the chain structure [38], exposes frequency of node visits during transactions, inability to store huge data on chain and high storage cost. Ancile and Medrec has scalability issues that resolves by our framework contribution of using IPFS by providing secure data storage in the offchain instead storing on the chain itself. FHIR chain proposed by Zhang et al [30] aims at secure sharing of clinical data by employing the Ethereum blockchain in which the onchain stores only encrypted metadata that serves as a pointer to the original healthrecords, whereas the original medical data is stored in the off chain database. Dubovitskaya et al [39] proposed a permissioned blockchain for secure data sharing focused on oncologic care that leverages local database and cloud services to store encrypted patients’ data. However, this approach also makes use of an arbiter for uploading the data in the cloud which makes the system less patient-centric. Another approach proposed by Wang and Song [31] is a secure cloud based EHR system using attribute based encryption and blockchain for the secure sharing of medical data. This approach includes the hospital as an arbiter for encrypting patients’ data which again contradicts the decentralized advantage of blockchain technology and makes it less patient-centric.

There are some techniques that used blockchain technology for sharing healthcare information including EMR and PHR but still failed to address data storage and efficient sharing of health data [32]. Another secure cloud blockchain EHR system proposed by wang and song based on attribute based cryptosystem integrating identity-based encryption and digital signatures [31]. Another IoT based blockchain platform was presented for tracking patient vital signs using smart blockchain based smart contracts [33]. Andrea et al. proposed a provenance management platform for tracking electronic healthcare records by employing Hyperledger Fabric blockchain smart contracts [34]. A.Roehrs et al. [35] presented a prototype implementation and evaluation of the OmniPHR architecture that maximizes the replication of health data across computing nodes model by integrating distributed health re-cords using blockchain technology and the open EHR interoperability. Another advanced decentralised privacy preserving technique was proposed for remote patient monitoring based on Internet of Things (IoT) based technology [36]. Several techniques have also been proposed to achieve computational power by employing neural networks [40–42]. Most of the existing approaches fail to guarantee all the essential requirements such as data privacy, security, secure storage, efficient access control, scalability and interoperability for EHRs. Our research work unravels most of the existing challenges in the e-health environment by employing a permissioned blockchain framework by utilizing Practical Byzantine Fault Tolerance(PBFT) [43] as consensus to enable data sharing in a decentralized fashion via IPFS by maintaining effective patient privacy, confidentiality and integrity for health records.

## 3 Preliminaries

### 3.1 Components of healthchain framework

A brief explanation of the preliminary components of our proposed Healthchain framework are outlined as follows:

#### 3.1.1 Membership service provider

Membership Service Provider (MSP) [20] abstracts away all the cryptographic mechanisms such as identity validation, signature generation and verification, protocols behind issuing and validating certificates and user authentication in the healthchain. The default interface for MSP used in this model is Fabric-Certificate Authority (CA) API and there is flexibility for the participating organizations to implement an External CA.

#### 3.1.2 Consensus mechanism

One key property and fundamental layer of blockchain is the consensus mechanism for transactions which depends on smart contracts layer to validate and update transactions in the ledger in accordance with the order they occur. Consensus protocol determines the order of transactions and rejection of bad transaction in the ledger. Practical Byzantine Fault Tolerance(PBFT) [16] is the employed consensus in this framework that utilizes crash fault tolerant or Byzantine Fault tolerant and do not require mining to accomplish consensus.

#### 3.1.3 Hyperledger fabric

Hyperledger Fabric [16] is the first permissioned blockchain platform that features a modular architecture established by IBM under Linux foundation for distributed ledger solutions. This research employs Hyperledger Fabric as the permissioned blockchain framework composed of pre-specified parties for sharing the healthcare information in a reliable way without any central authority. The biggest advantages of this research in developing using Hyperledger fabric is that it uses Byzantine Fault Tolerant consensus protocol [44] that does not entail mining or an associated currency to achieve consensus.

#### 3.1.4 Couch DB

CouchD and LevelDB are the two types of peer databases supported by Hyperledger Fabric. LevelDB is the default state database embedded in the peer nodes and stores chaincode data as simple key-value pairs and supports key, key range, and composite key queries. CouchDB [16] is a JSON format datastore instead of a pure key-value store that allows information mapping of the database documents. CouchDB is the on-chain database used in this research that can also improve compliance security and data protection in the healthchain.

#### 3.1.5 Hyperledger composer

Hyperledger Composer [45] is a set of collaborative tools for the designing and modelling of blockchain business networks that makes it easy and quick to build simple smart contracts and blockchain applications for business owners and developers. Composer, in this research creates a business network definition comprised of model file(.cto) that define the assets, script file(.js) with associated smart contracts, ACL(.acl) for access control rules and permissions and Query(.qry) files for defining queries to query the state database in the healthchain framework. Moreover, it packages the business network definition to a .bna file for deploying the healthchain business network to a distributed ledger.

#### 3.1.6 SmartContracts- chaincode

Smart contracts are self-executing chain codes that encodes the rules of certain network transactions, and are currently written in Go language that is installed and instanced by authorized participants on channel peers. This research work uses smart contracts that encompass the application logic of the system for EHR transactions particularly for data transmission, access management, request handling such as update medical records, allow doctors write, ereferrals to other doctor, update ownerships, eprescription to pharmacist [46]. Smart contracts will be executed during user interaction to identify request, validate request and for granting access permissions, update permissions for medical records.

#### 3.1.7 Interplanetary file system

IPFS [47] is a peer-to-peer distributed file system that shifts the present version of web to a distributed version and it can be used to replace HTTP. For example; if we want to retrieve a data structure or download a file that is available on the web using IPFS it can be retrieved through the peers in the network using a ‘cryptographic hash’ or unique fingerprint of that file by using content addressing property of IPFS. IPFS stores the encrypted data in multiple nodes if the data is higher than a defined threshold (size>256KB). In the context of this research, IPFS is used as an off-chain database for the storage of infinite healthcare records in which the medical records are encrypted using public key encryption before storage and hash of the health records will be stored in couch database.

## 4 Proposed framework

### 4.1 Overview of proposed framework

**Fig 1:**
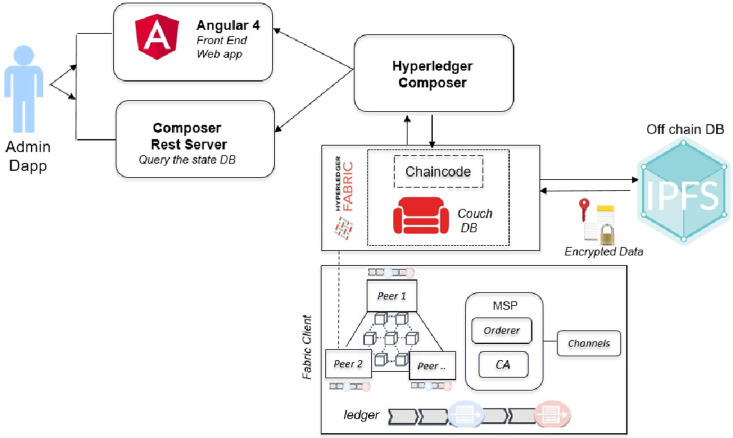
Healthchain architecture.

The proposed Healthchain architecture is shown in Fig 1. This framework includes Angular 4, Composer Rest Server, Hyperledger Composer, Hyperledger Fabric, Chaincode, CouchDB, IPFS and Fabric Client. Angular 4 is the Front end of the DApp (decentralized application) framework that connects with Composer Rest server which exposes and visualizes the state database, couchDB. The DApp admin interacts with user interface via Angular framework and the application processes user requests to the fabric network through a REST API known as the composer Rest Server. The REST API is used to retrieve the current state of the on-chain database which is the couchDB wherein the Angular framework retrieves the data through GET calls to the composer Rest API. Hyperledger composer builds and models the blockchain business network to create smart contracts for decentralized applications. Hyperledger Fabric [16] is the permissioned blockchain platform for distributed ledger solutions that supports the development of smart contracts known as chaincodes which is writable in Go, Java and Node.js to validate medical data entries by network participants. Healthchain framework employs a two-pronged solution platform (1)on-chain solution implemented on the secure network of Hyperledger Fabric utilizes the on-chain database Couch DB (2)off-chain solution to securely store data via IPFS (Interplanetary File System). Similar to Bitcoin [11] designed to maintain financial transactions, healthchain is intended for transactions in the healthcare that is secured via cryptography. In Healthchain, any interactions with the health records will be recorded as a transaction on the network and the transactions will be visible only to the participants related to the transaction.

**Fig 2:**
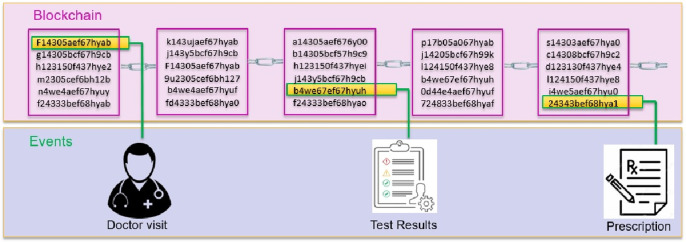
Overview of healthchain.

**Fig 3:**
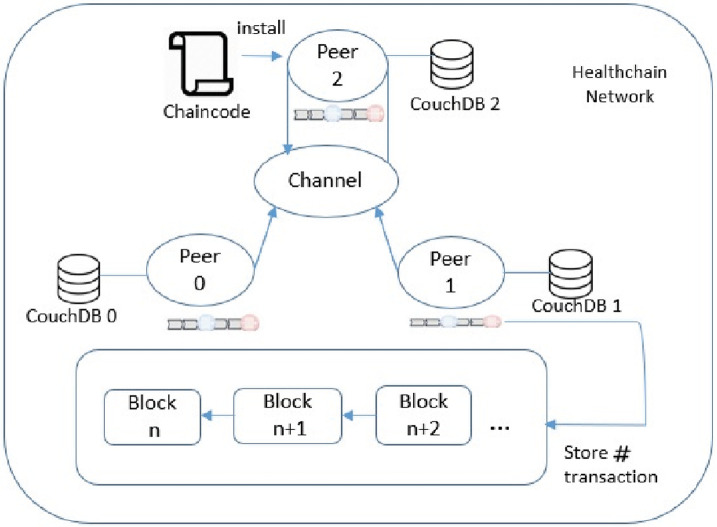
Nodes in healthchain.

Overview of the healthchain is shown in Fig 2. It shows a log of transactions as hash values in the blockchain for every event occurred in the healthcare such as a record creation, access, modification or updation. From Fig 2 it is evident that each transaction has a unique hash that guarantees the integrity of the health records and allows append-only revisions. Moreover, it produces a different hash which will not match the prior hash if the record has been tampered. When the identity management is combined with blockchain applications, the ledger becomes the supreme indicator of who did what and when on a blockchain. The working prototype is implemented on a permissioned blockchain called Healthchain on Hyperledger Fabric by employing Hyperledger Composer to create decentralized web applications for a single organization by incorporating three peer nodes as shown in Fig 3. This organization has three peer nodes with one anchor peer node as validating node and an ordering node (Kafka) with a single public channel for registering the network participants. System contains multiple peer nodes configured to use corresponding CouchDB as the world state database and IPFS as the distributed database, a solo ordering node, a Certificate Authority, Membership Service Provider (MSP) and Smart contracts for connecting to the blockchain. This can be extended to multiple peer nodes and multiple organisations in different machines to prove the system scalability. This framework has ledgers and associated smart contracts which has access to the ledgers. The application connects with peer nodes that invokes smart contracts to update the ledger. The Hyperledger Fabric healthchain network is built in a single organisation with three peer nodes using docker containers on the local computer but clearly, in the real world, it would be in separate IP networks or protected cloud environments. The organisation’s three peers are labelled as peer0 (P0), peer1(P1) and peer2 (P2) in which each holds their own instance of ledgers and copies of smart contracts. A single channel is designed so that Hyperledger Composer can communicate peers via the channel. In this network, our application A1 generates a transaction T1 to peers peer0, peer1 and peer2 via Channel C. Whenever a transaction executes, the chaincode will be installed to the peers. Application interacts with Peers and invokes chaincodes for querying or modifying the ledger. The transactions are stored within the blocks as hash values in the blockchain enables the history of changes that contributed to the healthchain framework. A block in the ledger pertaining to the health record of a patient i mainly comprises of the workload of that transaction W_*t*(*i*)_, hash of the previous transaction Wp_#(*i*)_ and hash of the current transaction W_#(*i*)_. The total workload of that block can be calculated as W_*Tot*(*i*)_:

$$\begin{array}{c} W_{Tot}\left(i\right)=W_{t}\left(i\right)+Wp_{\#}\left(i\right)+W_{\#}\left(i\right) \end{array}$$ (1)

### 4.2 Cryptographic process in healthchain

**Fig 4:**
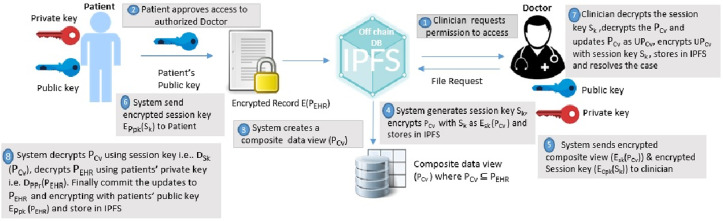
Cryptographic process in healthchain.

**Fig 5:**
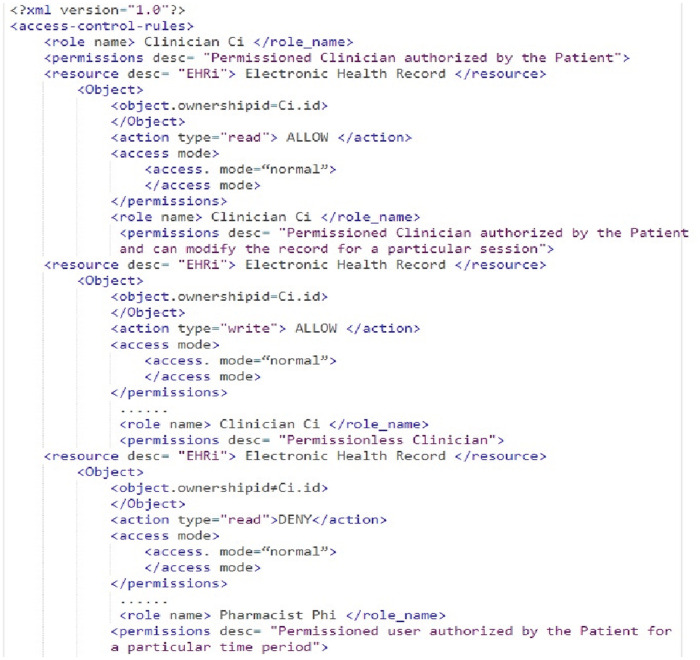
A snippet of the xml document showing access control permission rules.

**Fig 6:**
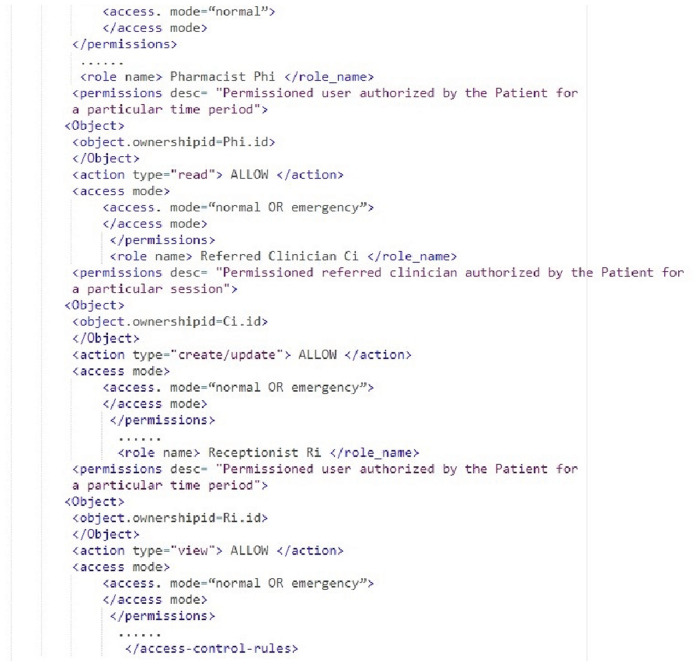
Access control permission rules for healthchain network.

Blockchain systems leverage cryptographic techniques to ensure the data integrity and confidentiality. This research employs special public key cryptography for encrypting the data in the off chain storage, IPFS. The wholistic view of the patient-doctor interaction for accessing health records is outlined as shown in Fig 4. The clinician (Doctor) requests permission to access the health record of the patient stored in the IPFS. The patient approves or grants the request from permissioned users on the basis of role and rule based access control permissions as shown in Figs 5 and 6. System in this framework refers to client-side application. The system generates a composite view of the record on basis of the request, alternately sharing the whole patient data. The system further generates a session key S_*k*_ to access records for a definite session and encrypts the composite view with the session key and then stores in IPFS. The system will also send the encrypted session key and encrypted composite view to the clinician. Besides, the system also shares the encrypted session key with the patient. The clinician decrypts the session key, decrypts the composite view and updates the composite view as updated record. Further, the clinician resolves the instance after encrypting the updated record with session key and uploads to the IPFS. The system notifies the record updates to the patient. The system decrypts the updated composite view using the session key, decrypts the encrypted medical record with patient’s private key from the IPFS. Finally, the system commit the updates to the original record, encrypts the original record with public key of the patient and upload it to the IPFS. The session key and the composite view for each session expires on session completion. The procedure can be explained with detailed notation in the following algorithms:

### 4.3 Proposed algorithms

Table 2 depicts the explanation of notations used in the algorithms and Algorithm 1 presents the algorithm to create and update health records by clinician in the healthchain. In our Healthchain framework there are 4 stakeholders in which P denotes Patient, C for Clinician, R for Receptionist and Ph for Pharmacist. We assume there are n participants for each stakeholders in the proposed framework. The Fabric-CA issues public key certificates to all n participants such as Patient, Clinician, Receptionist and Pharmacist. There will be a key pair for each participant in which $\text{P}{}_{Pk_{i}}$ and $\text{P}{}_{Pr_{i}}$ as the public and private keys of the patient P_*i*_, $\text{C}{}_{Pk_{i}}$ and $\text{C}{}_{Pr_{i}}$ as the public and private keys of clinician C_*i*_, $\text{R}{}_{Pk_{i}}$ and $\text{R}{}_{Pr_{i}}$ as the public and private keys of the Receptionist R_*i*_ and $\text{Ph}{}_{Pk_{i}}$ and $\text{Ph}{}_{Pr_{i}}$ as the public and private keys of the Pharmacist Ph_*i*_ respectively where i = 1 to n. This scenario gives a detailed explanation of how the Clinician and Patient interacts for accessing health records in the Healthchain framework. The Algorithm 1 is explained as follows. Consider that the patient P_*i*_ grants access to his/her medical record $\text{P}{}_{EHR_{i}}$ to clinician C_*i*_ upon request based on access control permissions as shown in Figs 5 and 6. The system then creates a composite view $P_{Cv_{i}}$ of the patient record $P_{EHR_{i}}$ that can be accessible to the clinician C_*i*_ on request alternately sharing the whole medical record of the patient. Composite view $P_{Cv_{i}}$ is the attribute set of the stored medical record $\text{P}{}_{EHR_{i}}$ that the system creates on permissioned user request without sharing the complete patient record. The composite view of a specific health record restricts access to the original data in such a way that a user can see and modify only selected data they need and no more. In other words $P_{Cv_{i}}$ is a subset of $P_{EHR_{i}}$ as shown in the Eqs (2) and (3).

**Table 2:** Explanation of notations.

Notations	Definition
IPFS	InterPlanetary File System
*P*_*Cv*_	Composite data view
S_*k*_	Session Key
C_*Pk*_	Public Key of Clinician
C_*Pr*_	Private Key of Clinician
P_*EHR*_	Patients’ Health record
P_*Pk*_	Patients’ public key
P_*Pr*_	Patients’ private key
P_*i*_	Patient
C_*i*_	Clinician
R_*i*_	Receptionist
Ph_*i*_	Pharmacist
U*P*_*Cv*_	Updated Composite view
U*P*_*EHR*_	Updated Health Record

**Algorithm 1** System(): Create and update composite view of Medical Records

 **Input**: A Clinician C_*i*_ with public key $\text{C}{}_{Pk_{i}}$ and session key S_*k*_ to access medical record $\text{P}{}_{EHR_{i}}$

 **Output**: Creation and updation of the medical record

1: **for** each user U with access permission to $\text{P}{}_{EHR_{i}}$

2: Algorithm checks xml access permission rules to grant or deny access to the user

3: **if** (permission type == “ALLOW” && role Type == ‘Clinician’)

4: Create composite view $P_{Cv_{i}}$ of the medical record $\text{P}{}_{EHR_{i}}$ in IPFS

5: $P_{Cv_{i}}\to \int_{i=1}^{n}(\text{D}{}_{P_{Pr_{i}}}(\text{E}{}_{P_{Pk_{i}}}(\text{P}{}_{EHR_{i}})))$

6: $P_{Cv_{i}}\subseteq \text{P}{}_{EHR_{i}}$

7: Generate a session key S_*k*_

8: ${\text{P}}_{i}\leftarrow \text{E}{}_{P_{Pk_{i}}}({\text{S}}_{k})$ /*Send encrypted session key to patient

9: ${\text{C}}_{i}\leftarrow \text{E}{}_{C_{Pk_{i}}}({\text{S}}_{k})$ /*Send encrypted session key to clinician

10: ${\text{C}}_{i}\leftarrow \text{E}{}_{S_{k}}(P_{Cv_{i}})$ /* Send encrypted composite view to clinician

11: **Algorithm 2()** /* Call Algorithm 2() for clinician record access and update

12: $\text{P}{}_{EHR_{i}}\leftarrow (\text{D}{}_{P_{Pr_{i}}}(\text{E}{}_{P_{Pk_{i}}}(\text{P}{}_{EHR_{i}})))$

13: $\text{U}P_{Cv_{i}}\leftarrow (\text{D}{}_{S_{k}}(\text{E}{}_{S_{k}}(\text{U}P_{Cv_{i}})))$

14: $\text{UP}{}_{EHR_{i}}\leftarrow [(\text{E}{}_{P_{Pk_{i}}}(\text{P}{}_{EHR_{i}}))+(\text{E}{}_{P_{Pk_{i}}}(\text{U}P_{Cv_{i}}))]$ /*System commits the update to the original record

15: **return** #

16: **else**

17: access ← deny

18: **return** access

$$\begin{array}{c} P_{Cv_{i}}\subseteq P_{EHR_{i}} \end{array}$$ (2)

$$\begin{array}{c} P_{Cv_{i}}=\left(D_{P_{Pr_{i}}}\left(E_{P_{Pk_{i}}}\left(P_{EHR_{i}}\right)\right)\right) \end{array}$$ (3)

The system further generates a session key S_*k*_ shared between clinician and the patient for a definite session. The system then sends the encrypted session key S_*k*_ to the patient as $E_{P_{pk_{i}}}$(S_*k*_) and clinician as $E_{C_{pk_{i}}}$(S_*k*_) by encrypting using respective public keys of the patient $\text{P}{}_{Pk_{i}}$ and clinician $\text{C}{}_{Pk_{i}}$ for a distinct session as shown in step (8) and step (9) in Algorithm 1. The Composite view $P_{Cv_{i}}$ will also be encrypted with session key S_*k*_ as $E_{S_{k}}(P_{Cv_{i}})$ and stores in IPFS. In addition, the system sends encrypted composite view $E_{S_{k}}(P_{Cv_{i}})$ to the clinician. Here the Algorithm 1 calls Algorithm 2 for clinician update of health records. Now, Clinician decrypts the session key with his private key and decrypts the composite view with the session key as shown in step (2) and step (3) in Algorithm 2. If there are any updates, clinician updates $P_{Cv_{i}}$ as $\text{U}P_{Cv_{i}}$, resolves the case, encrypts with the session key and uploads $\text{U}P_{Cv_{i}}$ to IPFS as $E_{S_{k}}(\text{U}P_{Cv_{i}})$. System refers to the client-side application in this framework. The patient uses a pass code to encrypt the private key $\text{P}{}_{Pr_{i}}$ and stores it on the client side. Every time for convenience, the patient can provide this pass code that decrypts the private key instead of sharing or uploading the private key, and the client end application can use this private key to decrypt the medical record. On clinician’s record update calls Algorithm(1) in which the system decrypts the encrypted record ie. $E_{P_{Pk_{i}}}(P_{EHR_{i}})$ using patient’s private key and decrypts the encrypted updated composite view from the IPFS ie. $E_{S_{k}}(\text{U}P_{Cv_{i}})$ using the session key as shown in steps (12) and step (13) in Algorithm 1. Finally, the patient commits the updates to the original record and encrypts the original record $P_{EHR_{i}}$ as $E_{P_{Pk_{i}}}(P_{EHR_{i}})$ before uploading to IPFS as shown in Eq (4).

$$\begin{array}{c} UP_{EHR_{i}}=\left[\left(E_{P_{Pk_{i}}}\left(P_{EHR_{i}}\right)\right)+\left(E_{P_{Pk_{i}}}\left(U_{P_{Cv_{i}}}\right)\right)\right] \end{array}$$ (4)

The session key S_*k*_ and the composite view $P_{Cv_{i}}$ for each session expires on session completion. The transactions eventuated on clinician access and record updates that invoke smart contracts thus creates a unique hash value and added to the healthchain. This composed of two main algorithms as summarized in Algorithm 1 and Algorithm 2.

**Algorithm 2** System(): Algorithm for clinician creating and updating medical records in Healthchain

 **Input**: A Clinician C_*i*_ with public key $\text{C}{}_{Pk_{i}}$ and session key S_*k*_ to create medical record $\text{P}{}_{EHR_{i}}$

 **Output**: Record Creation and updation

1: **for** each clinician with access permission on receiving encrypted S_*k*_ and $P_{Cv_{i}}$

2: ${\text{C}}_{i}\leftarrow \text{D}{}_{C_{Pr_{i}}}({\text{S}}_{k})$ /*Decrypt session key with Clinician’s private key

3: ${\text{C}}_{i}\leftarrow \text{D}{}_{S_{k}}(P_{Cv_{i}})$ /*Decrypt composite view with clinician’s session key

4: $\text{P}{}_{Cv_{i}}\to (\text{U}P_{Cv_{i}})$ /* Clinician updates Composite view

5: $\text{IPFS}\leftarrow \text{E}{}_{S_{k}}(\text{U}P_{Cv_{i}})$ /* Encrypts updated composite view with Clinician’s session key

6: **System()** /*call System()

7: **End**

#### 4.3.1 Access control permission rules

Figs 5 and 6 shows a snippet of the xml structure of access control permission rules in Healthchain network. The Algorithm 1 checks the access management rules in Figs 5 and 6 for granting or denying access to the health records. Access control policies are defined to safeguard the privacy of patients’ healthcare records [48]. Algorithm 2 renders an algorithm for clinician creating and updating health records in the Healthchain network. When an access request is made, the algorithm verifies the access control rules that are written in extensible markup language in Figs 5 and 6 which defines the access rights of the user on resource EHR_*i*_ defined by the owner. This access rules will be stored in the blockchain and submitted to the blockchain channel through a transaction called Business Network archive Transaction. In this approach;

- the rules comprises of the condition specifying the ID of the subject to which the access control policy grants the right of access;
- conditions specifying sets of values authorized for the subject, resource, action type and environment attributes for the access to be granted.

In our framework, we designed the rules to modify these conditions properly when they transfer those access rights to other authorised users before submitting to the healthchain. The actors of this scenario is resource owner P, Resource EHR_*i*_ and several subjects such as C_*i*_, Ph_*i*_ and R_*i*_ in the healthchain framework. The clinician C_*i*_ or any user can only read, write, modify or update access to health records only according to the access control permissions. From the Fig 5 it is clear that if the subject id matches with the object ownership id and only if the subject is a permissioned stakeholder, permissions such as read, write access are allowed or otherwise access will be denied. The stakeholders such as Pharmacist and Receptionist in this healthchain framework has given read access only to composite view of the health records for a particular session if their subject id matches with the ownership id of the object or resource as shown in Fig 6.

## 5 Prototype implementation of proposed framework

This section gives a detailed description of how users and records are added in the healthchain framework; steps included to provide access permissions to authorized users; retrieval of records in the healthchain framework.

### 5.1 Adding users to the healthchain framework

**Fig 7:**
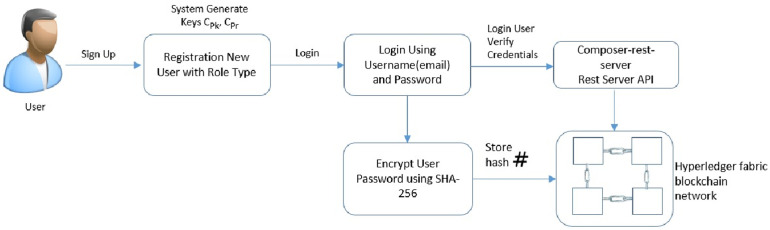
Adding users to healthchain.

The process for adding users to the healthchain network can be seen in Fig 7. The framework developed is role-based in which Patients, Clinicians(Doctors), Chemists and Receptionists can register themselves and login using login credentials such as email address and password. The nodes will be added by the network admin to the blockchain after validation from the consensus voter nodes. The patients’ and the users’ will be added to the healthchain with limited validation using their credentials such as username and password with each user having public private key pairs Pk_*i*_, Pr_*i*_. The user password is encrypted using SHA-256 hashing algorithm for improved security. The Composer Rest Server generates a REST API from the deployed blockchain business network that visualize and queries the values stored in couch database. The rest server also performs create, read, update and delete operations for assets and participants which allows transactions for processing and retrieval.

### 5.2 Adding records to the healthchain framework

**Fig 8:**
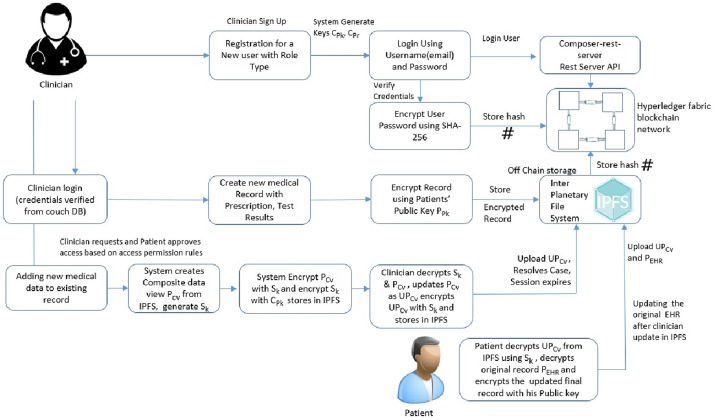
Adding records to healthchain.

Fig 8 shows the stage by stage process of how the Clinican adds medical record of the patient to Healthchain. This approach begins with assuming that the patient and the clinician have established an authorized relationship for updating health records. The process of adding medical records by clinician to the database is employed via internal encryption mechanism. There are two scenarios of adding patient records to the healthchain. (a)A new patient record will be created by the clinician to the healthchain through uploading the encrypted medical record using the patients’ public key to the IPFS. (b)A new patient record will be added or modified by the clinician; the system creates a composite view, $P_{Cv_{i}}$ of the data that can be accessible to the clinician C_*i*_ alternately sharing the whole data. The system further generates a session key S_*k*_ shared by patient and the clinician for a distinct session. The system then sends the encrypted session key S_*k*_ to the patient as $E_{P_{pk_{i}}}$(S_*k*_) and clinician as $E_{C_{pk_{i}}}$(S_*k*_) by encrypting using respective public keys of the patient $P_{Pk_{i}}$ and clinician $C_{Pk_{i}}$ for a distinct session. The Composite view $P_{Cv_{i}}$ will also be encrypted with session key S_*k*_ as $E_{S_{k}}(P_{Cv_{i}})$ and stores in IPFS. In addition, the system sends encrypted Composite view i.e.$E_{S_{k}}(P_{Cv_{i}})$ to the clinician. Now, Clinician decrypts the session key with his private key and decrypts the composite view with the session key. If there are any updates, clinician updates $P_{Cv_{i}}$ as $\text{U}P_{Cv_{i}}$, resolves the case, encrypts with the session key and uploads $\text{U}P_{Cv_{i}}$ to IPFS as $E_{S_{k}}(\text{U}P_{Cv_{i}})$. On clinicians’ record update, the system decrypts the encrypted record ie. $E_{P_{Pk_{i}}}(P_{EHR_{i}})$ using patients’ private key and also decrypts the encrypted updated composite view from the IPFS ie. $E_{S_{k}}(\text{U}P_{Cv_{i}})$ using the session key. Finally, the patient commits the updates to the original record and encrypts the original record $P_{EHR_{i}}$ as $E_{P_{Pk_{i}}}(P_{EHR_{i}})$ before uploading to IPFS. The session key S_*k*_ for each session expires and the composite view $P_{Cv_{i}}$ will be deleted after the session is completed. The transactions eventuated on clinician access and record updates will be hashed by employing smart contracts and added to the healthchain. This procedure can be summarized by two main algorithms as shown in Algorithm 1 and Algorithm 2 by employing Figs 5 and 6 for access management.

### 5.3 Providing access permissions to authorized users

**Fig 9:**
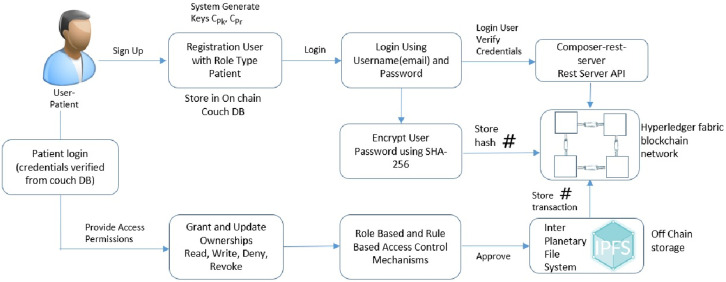
Providing access permission.

The patient has complete control and ownership to grant read, write, deny or revoke access permissions to the provider or other stakeholders such as receptionist, doctor or a pharmacist on the medical record thereby maintaining restrictive access control. Fig 9 describes the block diagram to provide access permission in the framework. The xml rules shown in Figs 5 and 6 presents read, write and deny access permission rules in the proposed healthchain. Moreover, the patient can permit access to health records based on authenticated user approved by the consensus in accordance with role type and permission type. Furthermore, the patient can also revoke the access from a particular clinician on his medical record and in that situation, the permission to the record can be denied from further access. As shown in Fig 9, Healthchain uses permission rules based on Role based and Rule based access control mechanisms for refined and restricted access to medical records. Smart contracts written will be executed during user interaction to identify request, validate request, updating records and granting access permissions for medical records.

### 5.4 Retrieval of records

**Fig 10:**
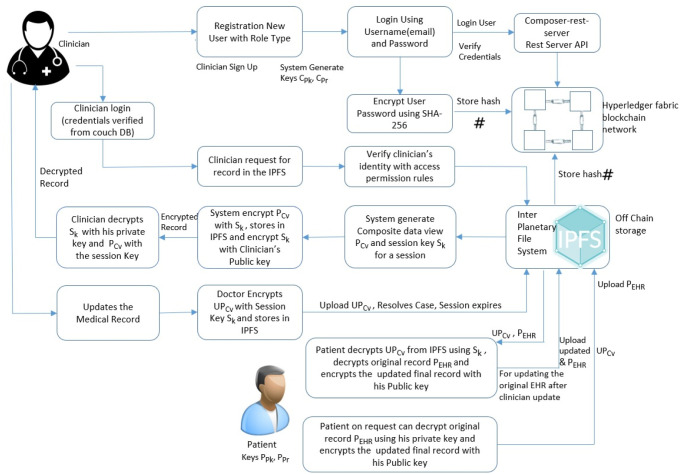
Retrieval of health records.

Retrieving a medical record can be performed through a series of transactions. The process begins with patient who uploads his data in IPFS via public key encryption. The clinician or a stakeholder who has access to the record for a particular session from IPFS, the system automatically generates a composite data view *P*_*Cv*_ which requires encryption with session key S_*k*_. Additionally the session key will be encrypted with the clinician’s public key C_*pk*_ for secure transfer. The clinician updates the medical record on arrival and encrypts with the session key before storing in the IPFS. The system notifies the patient regarding the updates on the medical record that decrypts the updated medical record $\text{U}P_{Cv_{i}}$ with the shared session key. The patient further encrypts the updated record with the patients’ public key, commits the updates to the original record and uploads to IPFS. Furthermore, the patient can decrypt his record using his private key from IPFS and upload the encrypted record using patients’ public key. All the transactions occured will be hashed by utilizing smart contracts and added to the healthchain. The step by step explanation is as shown in Fig 10.

## 6 Prototype implementation and results

For the implementation of our proposed Healthchain framework, we initially employed a private Hyperledger fabric blockchain viz healthchain in a Linux environment. Smart contracts are deployed for every transaction in the healthchain, IPFS storage system is utilized and network entities developed to build the healthchain framework. Following are the main components used for the simulation environment and Table 3 presents the machine configurations.

**Table 3:** Development environment for the proposed framework.

Component	Description
Operating Systems	Ubuntu Linux 16.04 64 bit
IDE	Hyperledger Composer
CPU	(Intel(R)Core(TM)i5-8500 CPU @ 2.5GHz 2.7GHz
Memory	8 GB
Node	v8.15.0
CLI Tool	Composer REST Server
Docker-compose	Version 18.09.2
Python	v2.7.12
Blockchain Network	Hyperledger Fabric
Framework Tools	Visual studio code
Programming Language	Angular 4,Node.js,composer modeling language
On-chain Database	CouchDB
Off-Chain Database	IPFS

The prototype is a user-centric model to process healthcare records using blockchain network, assuring the data ownership of individuals by preserving data security, privacy, data scalability and data integrity. This prototype is designed with few stakeholders namely Doctor (Clinician), patient, receptionist and pharmacist that builts a private healthchain framework. The framework’s flow is detailed as given below:

- Similar to a web application, URL of the framework is visible to users irrespective of the blockchain technology used at the rear end.
- The framework allows the user to signup with vital details like unique id, username, email address and password and the values will be stored in the onchain database, couchDB.
- The user can successfully log in if the username and password matches with the data stored in couch DB by querying the blockchain.
- A doctor logged in can upload the medical records to the IPFS by encrypting with the users’ public key thereby using public key encryption. The hash value generated by IPFS will be maintained in the couchDB, onchain database of the blockchain and thus preserves data integrity.
- A patient who is logged in, will be able to grant and deny accesses such as read, write, update permissions to the stakeholders on their medical records thus maintained restrictive access control.

**Fig 11:**
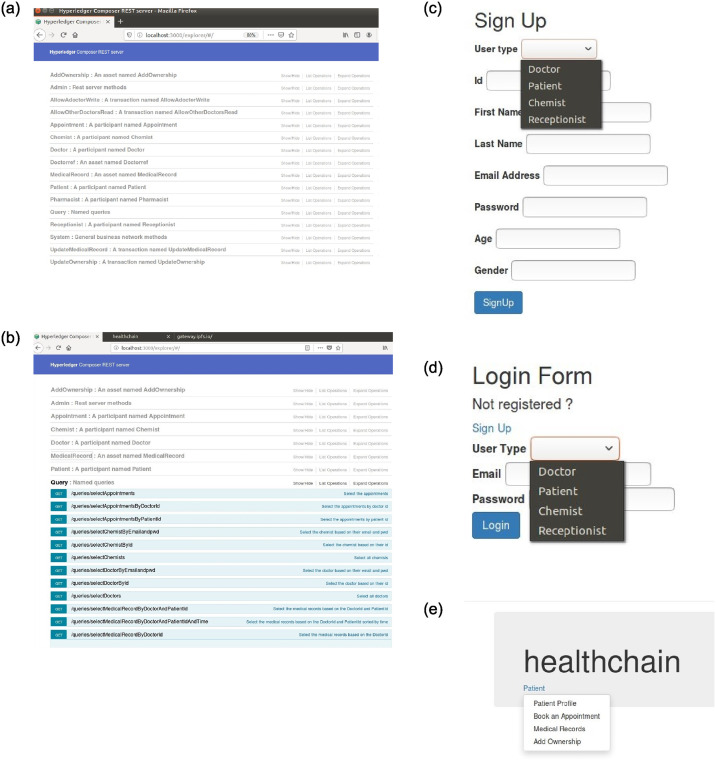
Illustration of EHR access in healthchain.

**Fig 12:**
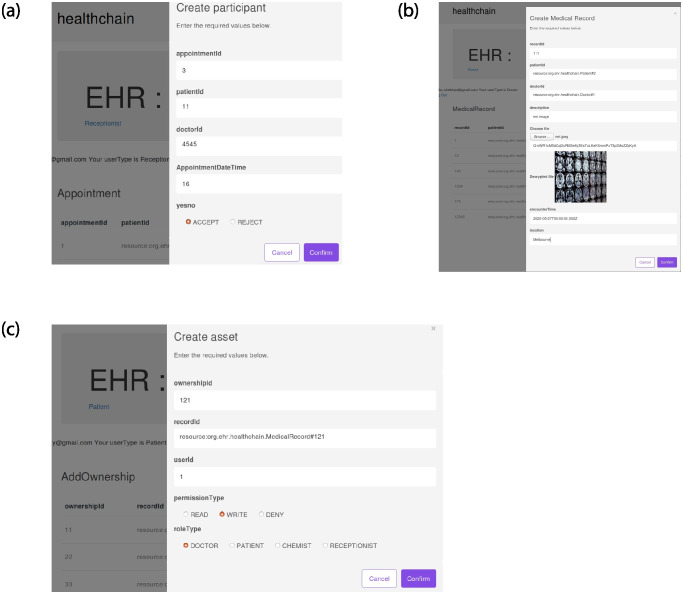
Illustration of EHR access in healthchain.

**Fig 13:**
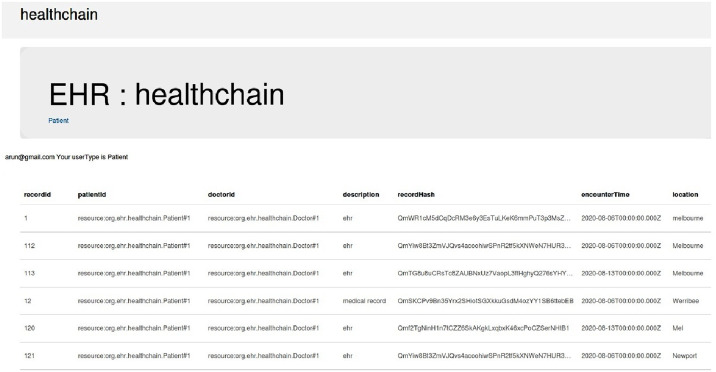
Illustration of provenance in healthchain.

**Fig 14:**
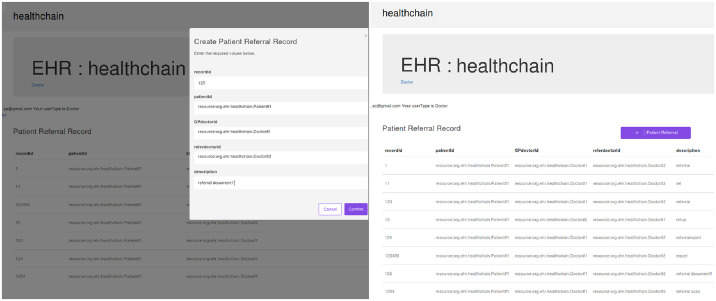
Illustration of referrals in healthchain.

**Fig 15:**
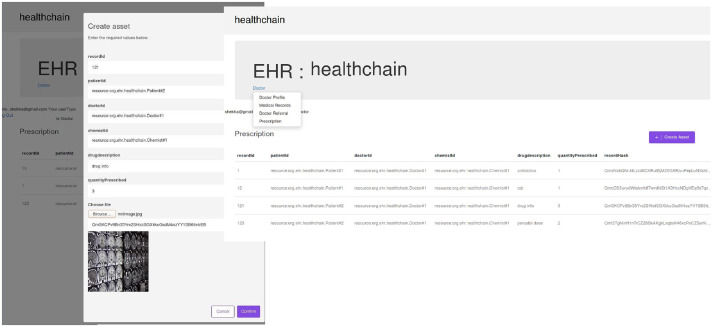
Illustration of creating prescription in healthchain.

The illustration of EHR access in Healthchain is presented in Figs 11–14 and 15. Fig 11(a) shows Rest API that exposes the CouchDB, state database of the blockchain. The data can be queried from the onchain state database via the Rest API as shown in Fig 11(b). Fig 11(c) is the User Sign Up in which the Patients, Doctors, Chemists and Receptionists can register in the healthchain using their roles.

After registration, the user can login with their email address and password by choosing their user type as shown in Fig 11(d). According to the role type Patient, the patient can view his profile, book an appointment for the doctor, view the medical records and add ownership to the doctor on his medical records as presented in Fig 11(e). The patient can book his appointment via Receptionist and the Receptionist can update the participant using patient id by accepting or rejecting the appointment as shown in Fig 12(a). After the approval of appointment by receptionist, the patient can consult the doctor and the doctor can create medical record for the patient. The clinical notes or the diagnosis results can be uploaded to IPFS using public key encryption for a session and IPFS returns the hash of encrypted record which is stored in the couch DB i.e. blockchain as illustrated in Fig 12(b). Being a patient centric blockchain, patients can also provide access permissions such as read, write and in certain situations where in the patient wants to revoke access to a doctor on his medical records, permission to the record can be denied as seen in Fig 12(c). Moreover, the patient can view the medical records added by the doctor as a data provenance [49] shown in Fig 13. Also, healthchain contributes secure e-referrals between doctors and doctor to Pharmacist interactions for drug tracking transaction via smart contracts in this research as shown in Figs 14 and 15. Fig 14 shows the process in which the Doctor creates patient referral records by using unique attributes and the corresponding view of referral records in the Healthchain. Fig 15 shows the representation of creating prescription in Doctor’s profile and resultant prescription view in healthchain which offers a promising solution to drug tracking that not only makes prescriptions safer but also guarantees a reliable transaction history of medical records.

## 7 Analysis of the framework

To validate the functional capability and to evaluate the performance of the prototype, some test cases have been explored. Four case studies are investigated to assess performance of the Healthchain framework systems which are illustrated in terms of efficiency, storage, security and scalability.

- Case I: Efficient storage of Health Records
- Case II: High Degree of Security
- Case III: Enhanced data privacy
- Case IV: Improved data scalability

### 7.1 Efficient storage of health records- case I

**Fig 16:**
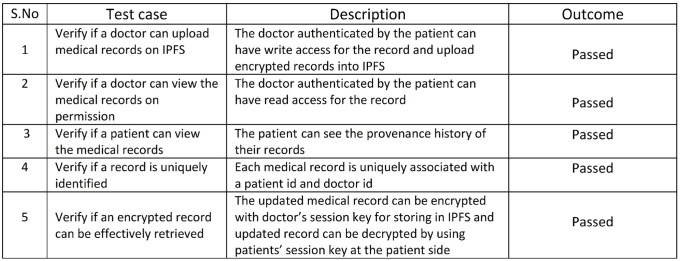
Storage of health records.

Efficient storage of health records in Interplanetary file system has been tested against a few cases listed in Fig 16. The first test case verifies if a doctor can upload health records or diagnosed test results on IPFS. The implementation results shown in Fig 12(c) shows that the authenticated doctor can have write access for the medical records and upload encrypted records into IPFS. A public key encryption algorithm has been used for encrypting the medical records on to the decentralized storage IPFS. The second case is tested if a doctor has read access permission to the medical records and is successfully verified as the doctor has been authenticated by the patient. Furthermore, it tests that a patient can view the medical records and Fig 13 portrays the provenance history of the medical records. Moreover, the system is tested against whether a record can be uniquely identified or not and has been successful as every medical record is uniquely related with a doctor id and patient id. Additionally the system has been checked against if an encrypted record can be effectively retrieved after decryption and has been successful as shown in Fig 12(b). The outcome is successful as the updated record can be encrypted with doctor’s session key for storing in IPFS and updated record can be decrypted by using patients’ session key at the patient side.

### 7.2 High degree of security- case II

**Fig 17:**
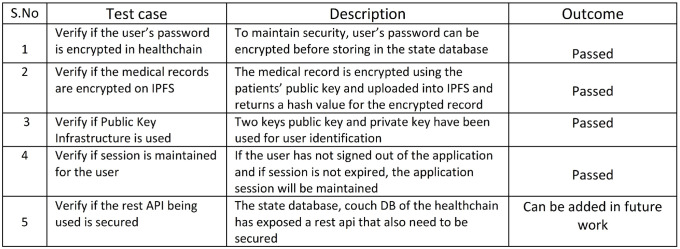
Degree of security.

Degree of security in healthchain has been verified against a few test cases as shown in Fig 17. The first case is tested and successful as the users’ password is encrypted before storing user authentication information in to the couch database. The second test case verifies the degree of security to check whether the medical records are encrypted on the IPFS and returns a unique hash for the encrypted record as shown in Fig 12(b). The outcome is favorable as the medical records are encrypted using the patients’ public key before uploaded into the IPFS. Furthermore, the prototype has also been verified with the usage of public key infrastructure and found successful since public and private keys are used for user identification. The prototype has also been tested to check whether the session has been maintained and found successful as long as the user has not signed out from the application and the session is not expired.

### 7.3 Enhanced data privacy- case III

**Fig 18:**
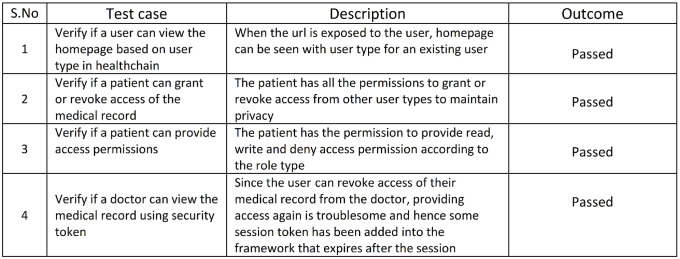
Enhanced data privacy: Access control.

HeathChain employs several privacy preserving mechanisms. The Data Privacy in healthchain is determined based on the permission to access the healthcare records. The access control for the medical records are tested against a few test cases as listed in Fig 18. The initial case is verified and successful as the users can view the homepage based on their user type as shown in Fig 11(e). Additionally the system has been tested to check whether a patient can provide grant or revoke access of the health records to the stakeholders and has been successful that preserves the data privacy. Furthermore, the system is also tested to see whether the patient can provide access permissions to the stakeholders. From the simulation results, it can be seen that patients can also provide access permissions such as read, write, and in certain situations where in the patient wants to revoke access of a doctor on his medical records, permission to the record can be denied as shown in Fig 12(c).

### 7.4 Improved data scalability- case IV

**Fig 19:**
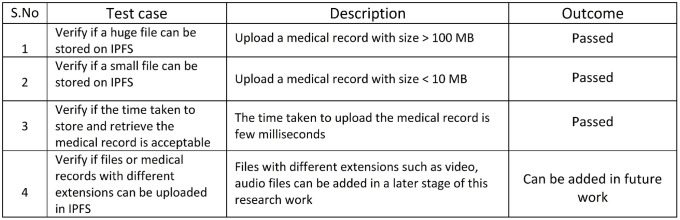
Improved data scalability.

Healthchain is well-founded on various notions to promote scalability. This research further contributes to data scalability by storing the hash value of medical records on chain and encrypted data off chain, in the decentralized storage, IPFS. Scalability of data has been examined against a few test cases as shown in Fig 19. A record of 100 MB was uploaded at a time to IPFS and has been successful which determined the scalability of the system. Considering the machine configuration, the system also verified that the average time taken by multiple users for the uploading and retrieval of the record was less than 60 seconds. A detailed view is portrayed in Figs 27 and 28. Therefore, it can be concluded that the system is able to handle a large dataset at low latency.

### 7.5 Comparative analysis of proposed framework with existing blockchain techniques

This section performs a comparative analysis of the proposed framework with the existing blockchain techniques in terms of major privacy preserving requirements viz Data Integrity, Data privacy, Data security, Confidentiality and Scalability. The proposed framework is compared against the existing blockchain based implementations such as [26, 31, 32] and [36]. From Table 4, it is evident that proposed system satisfies the shortcomings of existing systems in terms of data security, privacy and scalability.

**Table 4:** Comparative analysis.

Scheme	Data Integrity	Data Privacy	Data Security	Confidentiality	Scalability
MedChain [26]	✔	✘	✔	✔	✘
Wang & Song [31]	✔	✘	✔	✔	✘
Blochie [32]	✔	✔	✘	✔	✘
Blockchain for IoT [36]	✔	✔	✔	✔	✘
Proposed Framework (Healthchain)	✔	✔	✔	✔	✔

This section also describes how the proposed framework satisfies the privacy preserving requirements.

#### Data integrity

Data is immutable and tamper proof as the data is stored as hash values in each block and each block stores the hash value of the previous block in this blockchain framework. The trust on this blockchain framework is based on the consensus, digital signature and the designed cryptographic algorithm despite relying on a third party provider. Since all the blocks are linked, any alteration in the original data will result in a change in its hash value and it is computationally difficult to tamper the ledger, such that the non-tampering of the medical record is also explicitly guaranteed. In addition, the original data is stored in IPFS after performing a special cryptographic encryption technique and IPFS stores the data in multiple nodes if the size of data is higher than a defined threshold.

#### Data privacy

This framework provides a paramount significance to health record data privacy and Patient Privacy. Besides special encryption mechanisms that ensures data security, access control permission rules has been implemented in the system to safeguard the data privacy of patient health records. The framework ensures fine grained access control by integrating Role, Rule and Attribute based access control permission rules for any data request. Secondly, unauthenticated data requester cannot access data location since the blockchain stores only hash value of the encrypted medical record. Thirdly, if the data requester attributes do not meet the access policy embedded in the network archive file, it is also impossible to acquire any real medical record data from the blockchain public information.

#### Data security

Data Security is a crucial feauture as the EHR is cryptographically stored and dealt in the proposed system. This blockchain framework stores only hash of the encrypted data in the on chain and actual huge data is stored after encryption in the offchain storage. Since the framework is a patient centric approach that provides authenticated access permissioned by the patient guarantees the security of the health records. Also the smart contracts functionality combines with blockchain solutions embraces high-level encryption and ensures patient confidentiality in their health care information. In addition, the data stored on IPFS is encrypted using a special cryptographic algorithm to establish robust blockchain data solutions.

#### Confidentiality

In this framework, every health record of the patient will be stored in the IPFS after encrypting with patients’ public key and allows only the permissioned or authenticated users to access the record for a particular session. Since the framework is a patient centric approach in which the patient has complete control, unless for emergency situations to provide access permissions to the stakeholders, the confidential nature of health data is preserved.

#### Scalability

The proposed scheme preserves most of the privacy requirements and provides cryptographic storage of healthrecords in IPFS thereby resolves the scalability issue in the existing techniques. The scalability of the proposed system has demonstrated and proved that the system is capable of processing large datasets at low latency as shown in Figs 27 and 28.

### 7.6 Performance analysis and discussion

The evaluation matrix for the framework is shown in Table 5 that specifies the stakeholders, functions and solved problems to facilitate privacy preservation requirements. The framework is user-oriented that handles efficient storage and transfer of medical records ensuring the data ownership of the individuals, patient confidentiality and data integrity. By adopting access control mechanisms, clients can manage their own private information without jeopardizing confidentiality. Meanwhile, each requisition and update from the stakeholders viz receptionist, doctor, patient and pharmacist are reflected in the couchDB, state database of the healthchain. The patients’ can handle the access control mechanism by granting or revoking access of the medical records to the stakeholders thereby, maintaining user and data privacy. Data security and patient confidentiality is attained by data storage using public key encryption that secures the user data.

**Table 5:** Evaluation matrix.

Stakeholders	Functions	Solved Problems
Patients	Provide Access Control; User Login; Encrypted Data Storage; Decentralisation, Data Provenance; Data Retrieval	Data Confidentiality; Authentication; Privacy; Data Scalability; Authorization; Non-repudiation; Data Integrity
Doctors	User Login; Data Storage with Encryption; Secure e-Referral; Data Retrieval; Prescription Management	Authentication; Confidentiality; Scalability; Non- repudiation; Integrity; Security
Pharmacists	User Login; Prescription Management	Authentication; Non- repudiation; Integrity
Receptionists	User Login; Appointments Management	Authentication; Confidentiality

**Fig 20:**
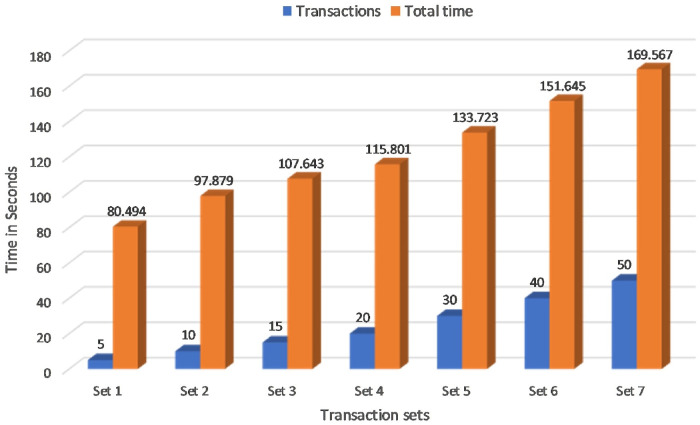
Transaction latency.

Several experiments have been carried out to analyse and evaluate the performance of the proposed blockchain system. The assets defined here are: (a)Medical Record (b)Referrals (c)Prescription (d)Add Ownership. Transactions are: (a)Create Medical Records (b)Update Medical Records (c)Allow Doctors Write (d)Update Ownership (e)eReferrals to other Doctor (f)ePrescription to Pharmacist. The first experiment calculated the Transaction Latency of the proposed blockchain framework as shown in Fig 20. Transaction Latency is the amount of time taken for the transaction to commit and available across the network nodes. if there are *n* number of nodes in the blockchain network, $\text{T}{}_{L_{n}}$ is the Transaction Latency, $\text{T}{}_{C_{n}}$ is the confirmation time in the network nodes and $\text{T}{}_{S_{n}}$ is the transaction submit time in seconds then;

$$\begin{array}{c} T_{L_{n}}=T_{C_{n}}-T_{S_{n}} \end{array}$$ (5)

**Fig 21:**
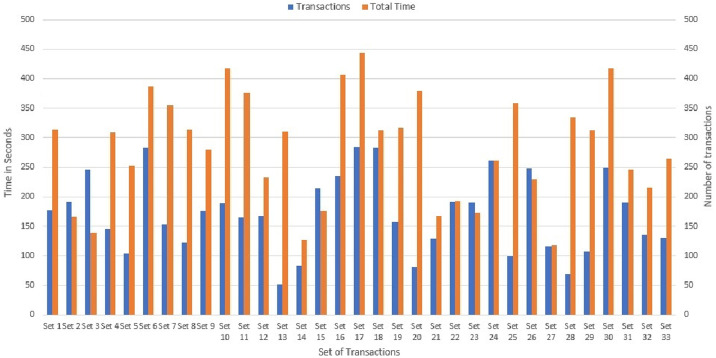
Transaction latency: Montecarlo simulation.

**Fig 22:**
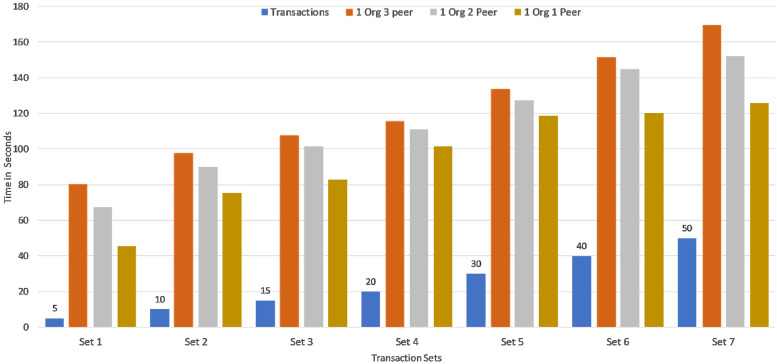
Transaction latency: Comparative analysis.

Seven sets of writing transactions to the network ledger were performed in various transaction sets within a range of 5,10,15,20,30,40 and 50 as shown in Fig 20. Considering the machine configuration in Table 3, it is clear that the initial set of 5 transactions took an average of 80 seconds to commit across the network and the final set of 50 transactions took an average of 160 seconds. The experimental result is further extended to Montecarlo Simulation environment for determining the transaction time for more number of transaction in the range of 50 to 300. It can be seen that average of 450 seconds was required to commit 300 transactions in three peer nodes as shown in Fig 21. Therefore, it is evident that the time taken to execute transactions increases with increase in peers and increase in the number of transactions. This Fig 22 shows a comparative analysis of Transaction Latency of 1 Org 1Peer, 1 Org 2Peer and 1 Org 3Peer. For seven sets of transactions ranging from 5, 10, 15, 20, 30, 40 and 50, It is clear that for 5 transactions, 1 Org 3Peer takes 80 secs to commit across the network in which 1 Org 2Peer took 67 secs to commit and 1 Org 1Peer took an average of 45 seconds to commit across the network. Therefore it shows that, more peers and higher number of organisations exhibit higher latency.

The second experiment calculated the Transaction Throughput of the proposed blockchain framework. The Transaction throughput is the rate at which the blockchain System Under Test (SUT) commits valid transactions in a defined time period at all network nodes. if there are *n* number of nodes in the blockchain network, $\text{T}{}_{T_{n}}$ is the Transaction Throughput, $\text{T}{}_{ct_{n}}$ is the total number of committed valid transactions in the network nodes and T_*tot*_ is the total time in seconds then;

$$\begin{array}{c} T_{T_{n}}=T_{ct_{n}}/T_{tot} \end{array}$$ (6)

**Fig 23:**
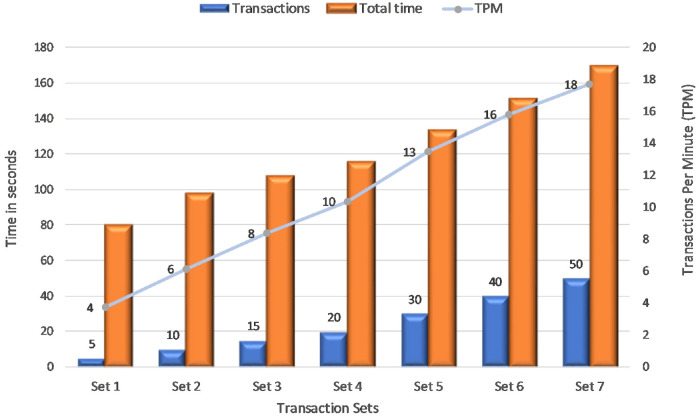
Transaction throughput.

This Fig 23 portrays Transactions Per Minute (TPM) for various sets of transactions. This experiment runs 7 sets of transactions to determine the TPM in the proposed system. The first set has 5 transactions, took approximately 80 seconds to commit in the network. As a result, the rate of valid transactions across the SUT is 4 TPM in the network. Similarly the last set of 50 transactions took approximately 160 seconds to be available across the network thereby can commit 18 TPM. x-axis indicates the transaction set, y-axis as time in seconds and secondary y-axis for TPM.

**Fig 24:**
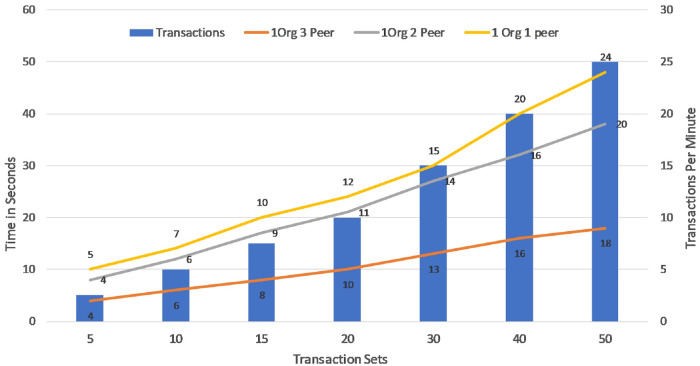
Transaction throughput: Comparative analysis.

Fig 24 demonstrates a comparative analysis of Transaction Throughput that calculates the TPM of the proposed framework for 1 Org 1Peer, 1 Org 2Peer and 1 Org 3Peer. From the Fig 24, It is evident that based on the Transaction Latency in Fig 22, the rate of valid transactions across the SUT is slightly higher for 1 Org 1Peer compared to 1 Org 2Peer and 1 Org 3Peer. The asset latency is the time taken by the SUT to successfully load and write the assets to the couchDB. if there are *n* number of nodes in the blockchain network, $\text{A}{}_{L_{n}}$ is the Asset Latency, $\text{T}{}_{Res_{n}}$ is the Response time and $\text{T}{}_{Sub_{n}}$ is the asset submit time in milliseconds then;

$$\begin{array}{c} A_{L_{n}}=T_{Res_{n}}-T_{Sub_{n}} \end{array}$$ (7)

**Fig 25:**
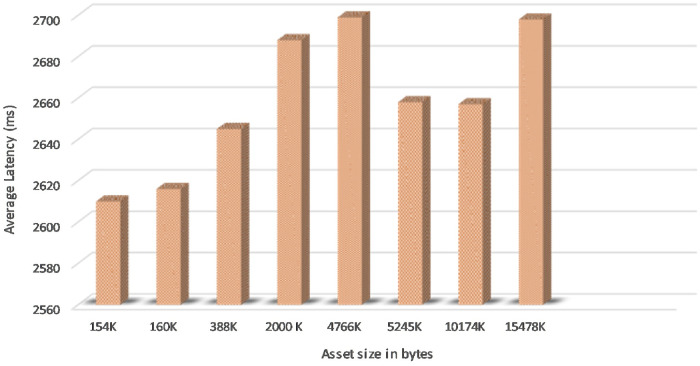
Asset latency.

Fig 25 shows the Asset Latency of varying assets size in bytes of 5 concurrent users in the proposed system and it is obvious that it took an average latency of 2.7 seconds to commit asset write updates in the couchDB across the network.

**Fig 26:**
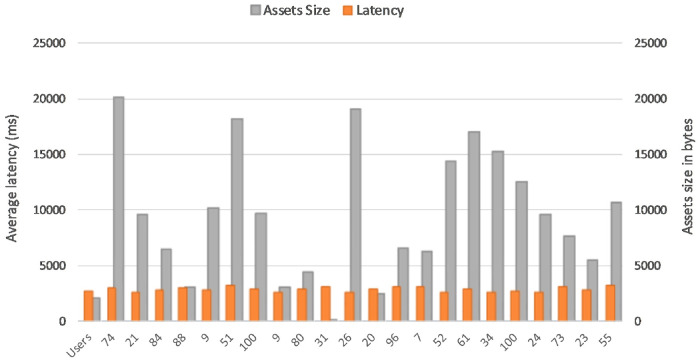
Asset latency: Montecarlo simulation.

**Fig 27:**
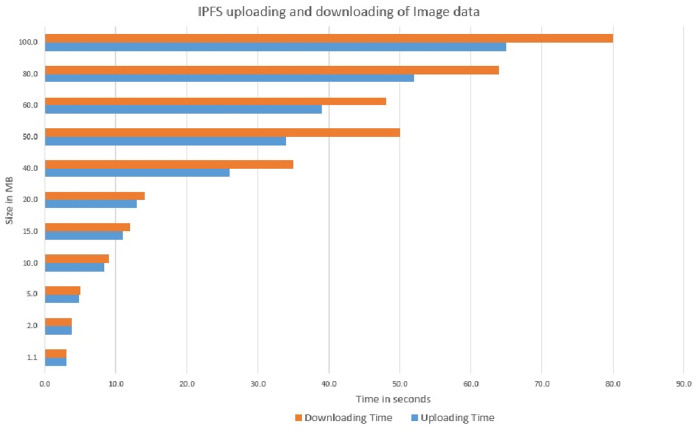
Uploading and downloading time comparison of image data in IPFS.

**Fig 28:**
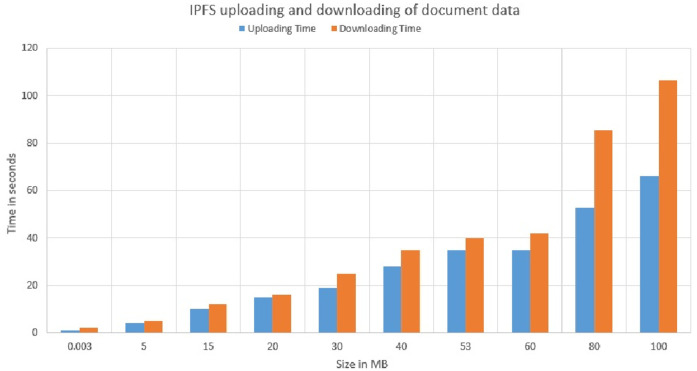
Uploading and downloading time comparison of document data in IPFS.

It is observed that asset size of 154K bytes took an average of 2.6 seconds and 15478K byte size took an average of 2.7 seconds to commit write updates in the CouchDB. We also extended the experiment to project the number of concurrent users in a range of 5 to 100 and byte size in a range of 154K bytes to 20574K bytes to determine the variation in Asset Latency through Monte carlo simulation and it took an average latency of 3.0 seconds to commit the asset updates in the ledger as shown in Fig 26. Considering the machine configuration in Table 3, system efficiency is higher as it is obvious that even if the number of users increases from 5 to 100 and assets size increases in the SUT, required a marginally small increase in time to commit the asset updates to the couchDB across the network. Scalability and efficiency have been achieved by uploading a record of 150 MB at a time to the IPFS and the average time taken for 5 concurrent users uploading and retrieval of the record was 60 seconds. Thereby, it can be concluded that the proposed system is capable of processing a large dataset at low latency. Data Provenance can also be attained via preserving user history in the blockchain thereby safeguarding Non-repudiation. Smart contracts combined with blockchain solutions embraces high-level encryption that allows the providers, users, patients and clinicians to ensure patient confidentiality in their health care information and enable it attack-proof. Furthermore, healthchain is designed to enhance scalability of healthcare data by storing hashes on chain and real data in the off chain IPFS. Figs 27 and 28 demonstrates the scalability of IPFS using both the image data and document data with a size comparison upto 100 MB size. The results are obtained from transaction execution of 5 users concurrently upload and download the data in IPFS. Considering the machine requirements, for a 100 MB image file, the system takes an average time of 65 sec to upload the data to IPFS and downloading in an average time of 80 seconds as shown in Fig 27. Also, the system takes an average of 65 seconds uploading time and an average time of 105 seconds for downloading a 100 MB document file as portrayed in Fig 28.

Healthchain is a Patient driven Interoperability framework and employs several security and privacy preserving mechanisms that sustain cyber attacks and internal attacks, however there are still some improvements that could be made to make it as a foolproof solution. Initially, the REST API can be made secure via using HTTPS by encrypting communications between client and server instead of HTTP that is being used nowadays. Secondly, we can employ smart contracts on a large scale that will be executing on more number of nodes for the privacy and safety of patients’ information to make it tamper resistant. This work can be extended to multiple nodes for proving the effectiveness of Distributed Ledger Technology in health records as a future work. The implementation of different smart contracts on every node and submitting the node to the system requires several stages of verification which is considered as a future work to prove the efficiency of the proposed system.

## 8 Conclusion

In this research work, a permissioned blockchain framework has been implemented for secure data storage and access of electronic health records utilizing Hyperledger fabric and Hyperledger composer. Since the blockchain is tamper resistant, the system is tamper proof to handle healthcare records that preserves data privacy, security and integrity. Moreover, no incentive mechanisms for blockchain mining are included that demonstrates the patients’ ownership towards their healthcare data. The research proposes an architecture for securing data storage and providing efficient access control between stakeholders viz patients, doctors, pharmacists and other participants via encryption techniques and access control mechanisms. Moreover, a working prototype based on Hyperldger Fabric and Interplanetary File System are made to illustrate the system’s viability. The proposed methodology has been implemented and evaluated with some use cases for EHRs and consequently, the framework is successful as a reliable health data network. The result of prototype implementation and analysis proves that the approach is a tamper resistant mechanism as information will be stored as hash values for every healthcare transactions in the blockchain. Moreover, it has enormous potential to ensure privacy, security, integrity, confidentiality and scalability of the e-health information. Performance evaluation of the proposed system is completed using empirical research for various scenarios by configuring asset size, block size, various nodes, asset creation time, transaction sets, for evaluation metrics such as Transaction Latency, Transaction Throughput, Asset Latency and Data Scalability for analysis. Furthermore, this research also explores technology framework and business processes for blockchain applications.

The introduction of this technological innovation which incorporates cryptographic elements offers a more secure and effective framework to store, transfer and access EHR in the cloud environment efficiently. The healthchain prototype based on the blockchain technology is a resilient tamperproof ledger from the test results and it rests heavily on the success. With increase in health data every year, we look forward to refine this prototype with rigorous simulations in scalability and comparing with other blockchain configurations in a test bed arena that will invite further attention in future research work.
